# Supplementary material for: Efficacy of Different Doses of Daprodustat for Anemic Non-dialysis Patients with Chronic Kidney Disease: A Systematic Review and Network Meta-Analysis
Source: J Clin Med. 2022 May 11;11(10):2722. doi: 10.3390/jcm11102722 (PMC9145143; doi:10.3390/jcm11102722)
Supplement: Supplementary file 1 [file jcm-11-02722-s001.zip › Supplementary Table S1, summary.pdf]

Table S1

Study ID	Study procedures				Inclusion criteria	Exclusion criteria	Length of follow up	Protocol registration	Primary outcomes/ endpoint	Conclusion
	Study arms	Sample size	Doses	Route of administration						
Brigandi 2016	Placebo	9	NA	oral	1) Male and female patients aged 18 to 85 years<br>2) Diagnosed with CKD (CKD-3/4/5 group: eGFR, 15-59 mL/min/1.73 m2 for stages 3-4	1) A positive pre-study Hepatitis B surface antigen or positive Hepatitis C antibody result within 3 months prior to Screening.<br>2) A positive test for HIV antibody<br>3) A pre-study drug screen that is positive due to drug use not associated with a current medication prescription.	57 days	NCT01047397	1) Rate of response for increase from baseline of hemoglobin levels<br>2) Adverse Events reporting<br>3) Safety Labs (Chemistry)<br>4) Vital Signs (blood pressure and heart rate)	"GSK1278863 induced an effective EPO response and stimulated non-EPO mechanisms for erythropoiesis in anemic non–dialysis-dependent and dialysis-dependent patients with CKD"
	Daprodustat	17	10mg	oral						
	Daprodustat	14	25mg	oral						
	Daprodustat	15	50mg	oral						
	Daprodustat	15	100mg	oral						
Holdstock 2016	Placebo	18	NA	oral	1) Age and weight: >/=18 years of age and >/=45 kg (weight post-dialysis).<br>2) On three times weekly hemodialysis for at least 8 weeks, irrespective of eGFR values and stage of chronic kidney disease (CKD).<br>3) A single-pool Kt/V urea of >/=1.2 based on a historical value obtained within the prior month in order to ensure the adequacy of dialysis. If Kt/V urea is not available, then an average of the last 2 values of urea reduction ratio (URR) of at least 65%.<br>4) rhEPO use: Using the same rhEPO (epoetins or darbepoetin) with total weekly doses that varied by no more than 50% during the prior 4 weeks (i.e., maximum vs. minimum total weekly doses </=50%).<br>5) Hgb concentrations 9.5-12.0 g/dL	1)Patients were excluded from the study if they were on dialysis or were expected to initiate dialysis during the time they would be in the study	4 weeks	NCT01587898	1) Modeled Hemoglobin (Hgb) Change From Baseline (Pre-dose on Day 1) at 4 Weeks of Treatment	“GSK1278863 was generally safe and well tolerated at the doses and duration studied. GSK1278863 may prove an effective alternative for managing anemia of CKD."
	Dabrodustat	17	0.5 mg	oral						
	Dabrodustat	18	2 mg	oral						
	Dabrodustat	19	5 mg	oral						
Holdstock 2019	rhEPO naïve, Daprodustat	123	2-4 mg	oral	1) Age: >=18 years of age.<br>2) CKD stage: Kidney Disease Outcomes Quality Initiative (KDOQI) CKD stages 3/4/5 defined by electronic estimated glomerular filtration rate (eGFR) using the CKD Epidemiology Collaboration (CKD-EPI) formula.<br>3) Hgb: Group 1 (rhEPO naïve): Baseline Hgb of 8.0-11.0 g/dL (inclusive) (USA sites only: 8.0-10.0 g/dL, inclusive); Group 2 (rhEPO users): Baseline Hgb of 9.0-11.5 g/dL (inclusive) (USA sites only: 9.0-10.5, inclusive).	1) Dialysis: On dialysis or planning to initiate dialysis during the study.<br>2) Renal transplant: Pre-emptive or scheduled renal transplant.<br>3) High rhEPO dose: An epoetin dose of >=360 IU/kg/week intravenous (IV) or >=250 IU/kg/week subcutaneous (SC) or darbepoetin dose of >=1.8 microgram per kilogram per week (mcg/kg/week) IV or SC within the prior 8 weeks through Day 1 (randomization).<br>4) Use of methoxy polyethylene glycol epoetin beta within the prior 8 weeks through Day 1 (randomization).	24 Weeks	NCT01977573	1) Mean hemoglobin levels	"Daprodustat effectively maintained target hemoglobin over 24 weeks in CKD patients with anemia who were rhEPO naive or had switched from existing rhEPO therapy"
	rhEPO naïve, Control	43	NA	NA						
	rhEPO user, Daprodustat	33	2 mg	oral						
	rhEPO user, Control	36	NA	NA						
Nangaku 2021	Daprodustat	149	2-4 mg	Oral	1) Adults with CKD stages G3, G4, and G5, ND in the previous 12 weeks, with hemoglobin ≥8.0 g/dL and <11.0 g/dL (ESA-naïve) or ≥9.0 g/dL and ≤13.0 g/dL (ESA users)	1) Kidney transplant: Planned living-related kidney transplant during the study Anemia-related criteria<br>2) Aplasia: History of bone-marrow hypoplasia or pure red cell aplasia<br>3) Other causes of anemia: pernicious anemia, thalassemia, sickle cell anemia, or myelodysplastic syndromes	52 weeks	NCT02791763	1) Mean hemoglobin concentration during weeks 40–52 (primary efficacy evaluation period)	"Oral daprodustat was noninferior to CERA in achieving and maintaining target hemoglobin levels in Japanese ND patients. Daprodustat was well tolerated, with no new safety concerns identified."
	CERA	150	NA	subcutaneous						
Singh 2021	Daprodustat	1937	2-4 mg	oral	1) Adults were eligible for screening if they had stage 3 to 5 CKD and were not currently receiving dialysis or scheduled to start dialysis within 90 days, met the hemoglobin and ESA criteria.<br>2) Had a serum ferritin level of more than 100 ng per milliliter and a transferrin saturation above 20%.	1) Patients who had anemia that was unrelated to CKD, a recent cardiovascular event, or current or recent cancer were excluded.	52 weeks	NCT02876835	1) Time to the first occurrence of adjudicated major adverse cardiovascular event (MACE) (composite of all-cause mortality, non-fatal myocardial infarction (MI) and non-fatal stroke).<br>2) Mean change in hemoglobin (Hgb) between baseline and efficacy period (EP)	"Among patients with CKD and anemia who were not undergoing dialysis, daprodustat was noninferior to darbepoetin alfa with respect to the change in the hemoglobin level from baseline and with respect to cardiovascular outcomes."
	Darbeopoetin Alfa	1935	NA	NA						
