# Supplementary material for: Efficacy of Different Doses of Daprodustat for Anemic Non-dialysis Patients with Chronic Kidney Disease: A Systematic Review and Network Meta-Analysis
Source: J Clin Med. 2022 May 11;11(10):2722. doi: 10.3390/jcm11102722 (PMC9145143; doi:10.3390/jcm11102722)
Supplement: Supplementary file 1 [file jcm-11-02722-s001.zip › Supplementary Table S2, baseline.pdf]

Table S2

Study ID	Study arm	Age (yr) (Mean ± SD)	Female sex N (%)	BMI, (Mean ± SD)	Hemoglobin — g/dl (Mean ± SD)	TIBC (mg/dL) (Mean ± SD)	Ferritin (ng/mL) (Mean ± SD)	TSAT (%) (Mean ± SD)	Hepcidin (ng/mL) (Mean ± SD)	Race					Baseline epoetin alfa dose (U/week) (Mean ± SD)	Patients receiving iron-containing phosphate binder, n (%)	Disease history, No. (%)				Prior ESA, No. (%)		CKD stage, No. (%)			Angina pectoris	Baseline eGFR (mL/min/1.73 m2), mean (SD)
										White N (%)	Black or African American N (%)	Other N (%)	Asian N (%)	Multiple N (%)			Diabetes mellitus	Hypertension	Dyslipidemia	Cardiovascular disease	Darbepoetin alfa	Epoetin beta pegol	**3**	4	5		
Brigandi 2016	Daprodustat 100 mg	62.1±12.38	-	-	-	-	-	-	-	-	-	-	-	-	-	-	-	-	-	-	-	-	-	-	-	-	-
	Daprodustat 50 mg		-	-	-	-	-	-	-	-	-	-	-	-	-	-	-	-	-	-	-	-	-	-	-	-	-
	Daprodustat 25 mg		-	-	-	-	-	-	-	-	-	-	-	-	-	-	-	-	-	-	-	-	-	-	-	-	-
	Daprodustat 10 mg		-	-	-	-	-	-	-	-	-	-	-	-	-	-	-	-	-	-	-	-	-	-	-	-	-
	Placebo	54.8±17.26	-	-	-	-	-	-	-	-	-	-	-	-	-	-	-	-	-	-	-	-	-	-	-	-	-
Holdstock 2016	Placebo	69.2±11	14 (78%)	32.1±9.6	9.91±0.57	49.1±4.3	243.7±161.1	22.1±7.5	432.3±226.6	12(63%)	4(21%)	1(5%)	2(11%)	-	16.7±7.3	-	12(67%)	17(94%)	14(78%)	18(100%)	-	-	4(23%)	8(44%)	6(33%)	18(100%)	23.1±9.6
	Daprodustat 0.5 mg	66.6±11.7	12 (71%)	34.2±9.7	9.98±0.58	53.4±7.7	265.5±235.7	21.6±7.4	298±145.63	10(63%)	4(25%)	0	2(13%)	-	13.05±5.5	-	12(71%)	16(94%)	11(65%)	17(100%)	-	-	5(30%)	9(53%)	3(18%)	17(100%)	23.8±9.8
	Daprodustat 2 mg	66.9±11.4	10 (56%)	60.6±5.5	9.74±0.7	51.8±9.5	350.5±266.4	25.5±6.4	306±107.8	12(67%)	4(22%)	0	2(11%)	-	15.03±8.53	-	14(78%)	18(100%)	13(72%)	18(100%)	-	-	5(28%)	7(39%)	6(33%)	18(100%)	24.2±12.5
	Daprodustat 5 mg	71.3±11.3	16 (84%)	31.2±7.6	10.08±0.72	56±10.3	298.6±264.8	25.6±7.5	384±218.7	14(78%)	2(11%)	1(6%)	1(6%)	-	13.68±6.13	-	12(63%)	19(100%)	17(89%)	19(100%)	-	-	6(31%)	8(42%)	5(26%)	19(100%)	24.2±10.8
Holdstock 2019	rhEPO naive Daprodustat	67.6±12.21	75(61%)	27.7±6.9	9.9±0.8	-	-	-	-	-	-	50(40%)	17(14%)	56(46%)	-	-	0	2(1%)	4(3%)	29(22%)	-	-	27(22%)	58(47%)	38(31%)	7(5%)	21.3±10.69
	rhEPO naive Control	64.3±14.22	23(53%)	28.4±5.4	9.9±0.73	-	-	-	-	-	-	16(37%)	6(14%)	21(49%)	-	-	0	0	2(4%)	12(27%)	-	-	7(16%)	17(40%)	19(44%)	5(11%)	19.4±109
	rhEPO user Daprodustat	62±14.06	17(52%)	28.1±7.1	10.2±0.62	-	-	-	-	-	-	15(45%)	9(27%)	9(27%)	-	-	2(6%)	1(3%)	1(3%)	9(25%)	-	-	4(12%)	17(52%)	12(36%)	0	17.9±9.17
	rhEPO user Control	66.7±12.89	23(64%)	27.3±6.4	10.2±0.57	-	-	-	-	-	-	9(25%)	9(25%)	18(50%)	-	-	0	0	0	6(17%)	-	-	4(11%)	15(42%)	16(44%)	3(9%)	18.8±11.9
Nangaku 2021	Daprodustat	68±12	53(36%)	23±3.4	10.5±1.1	-	159±89	-	-	-	-	-	-	-	-	30(28%)	65(44%)	141(95%)	91(61%)	12(8%)	25(17%)	33(22%)	15(10%)	74(50%)	60(40%)	12(8%)	-
	CERA	70±9	58(39%)	24±2.4	10.7±1.1	-	144±81	-	-	-	-	-	-	-	-	29(27%)	69(46%)	145(97%)	105(70%)	10(7%)	21(14%)	21(14%)	30(13%)	63(42%)	67(45%)	10(7%)	-
Singh 2021	Daprodustat	66.33±13.35	1102(56.9%)	27.13±5.86	9.9±0.9	45±7.42	295.67±216.62	30.33±9.64	111.07±77.3	1098(56.7%)	183(9.4%)	-	525(27.1%)	36(1.9%)	4241±2803	967(49.9%)	1076(55.5%)	1828(94.4%)	-	716(37%)	907(46.8%)	-	-	-	-	150(7.7%)	18.33±10.39
	Darbepoetin alfa	66±12.61	1071(55.3%)	26.97±5.71	9.8±0.9	44.33±6.68	298.33±206.24	29.33±9.64	112.1±80.57	1055(54.5%)	185(9.6%)	-	537(27.8%)	51(2.6%)	4242±2890	949(49%)	1118(57.8%)	1829(94.5%)	-	716(37%)	903(46.7%)	-	-	-	-	145(7.5%)	19±11.13
